# Internet-based medical education: a realist review of what works, for whom and in what circumstances

**DOI:** 10.1186/1472-6920-10-12

**Published:** 2010-02-02

**Authors:** Geoff Wong, Trisha Greenhalgh, Ray Pawson

**Affiliations:** 1 Research Department of Open Learning, Division of Medical Education, UCL, 4th Floor, Holborn Union Building, Whittington Campus, Highgate Hill, London N19 5LW, UK; 2 School of Sociology and Social Policy, University of Leeds, Leeds LS2 9JT, UK

## Abstract

**Background:**

Educational courses for doctors and medical students are increasingly offered via the Internet. Despite much research, course developers remain unsure about what (if anything) to offer online and how. Prospective learners lack evidence-based guidance on how to choose between the options on offer. We aimed to produce theory driven criteria to guide the development and evaluation of Internet-based medical courses.

**Methods:**

Realist review - a qualitative systematic review method whose goal is to identify and explain the interaction between context, mechanism and outcome. We searched 15 electronic databases and references of included articles, seeking to identify theoretical models of how the Internet might support learning from empirical studies which (a) used the Internet to support learning, (b) involved doctors or medical students; and (c) reported a formal evaluation. All study designs and outcomes were considered. Using immersion and interpretation, we tested theories by considering how well they explained the different outcomes achieved in different educational contexts.

**Results:**

249 papers met our inclusion criteria. We identified two main theories of the course-in-context that explained variation in learners' satisfaction and outcomes: Davis's Technology Acceptance Model and Laurillard's model of interactive dialogue. Learners were more likely to accept a course if it offered a perceived advantage over available non-Internet alternatives, was easy to use technically, and compatible with their values and norms. 'Interactivity' led to effective learning only if learners were able to enter into a dialogue - with a tutor, fellow students or virtual tutorials - and gain formative feedback.

**Conclusions:**

Different modes of course delivery suit different learners in different contexts. When designing or choosing an Internet-based course, attention must be given to the fit between its technical attributes and learners' needs and priorities; and to ways of providing meaningful interaction. We offer a preliminary set of questions to aid course developers and learners consider these issues.

## Background

The Internet is widely used in medical education [1]. Several previous systematic reviews and two meta-analyses have compared the efficacy and utility of Internet-based education with conventional teaching methods or no teaching [2-8]. Two main questions face researchers in this field: efficacy (*can *Internet-based medical education work, and if so what is the 'effect size' compared to conventional teaching?) and effectiveness (under what real-world circumstances does it *actually *work, and how might its impact and cost-effectiveness be maximised?).

Cook et al.'s 2008 meta-analysis addressed efficacy, and concluded that, on average, Internet formats were equivalent to non-Internet formats in terms of learner satisfaction and changes in knowledge, skills and behaviour [8]. Their findings indicated that substantial heterogeneity existed and their meta-analysis was unable to account for the complexity of the interactions within their included studies.

In trying to make sense of this heterogeneity we conceptualised educational courses as complex interventions and used the realist review method. Complex interventions consist of multiple human components (teachers, learners etc.) that interact in a non-linear fashion to produce outcomes which are highly context dependent [9-11]. Outcomes in such interventions depend on humans making decisions in a semi-predictable (demi-regular) manner about how to use the resources available to them in the context they find themselves in. Our rationale for using the realist review method is explained in the Methods section below.

## Methods

In this realist review we set out to supplement and extend previous systematic reviews and meta-analyses. In particular we sought initially to [a] explain what sort of Internet-based medical education 'works', for whom and in what circumstances; [b] produce pragmatic guidance that could be used by developers to optimise the design of their courses and by potential learners to evaluate whether a particular course is right for them; and [c] extend the methodological knowledge base in relation to secondary research in medical education.

### The realist review method

The realist approach to reviewing the evidence from complex interventions assumes that no deterministic theories can *always *explain nor predict outcomes in every context [12]. Instead it is based on the principle that, though human agency and interaction is involved, in certain contexts or situations, individuals are likely, though not always certain, to make similar choices about which resources they will use [13]. In other words, particular contexts influence human choice such that semi-predictable reoccurring patterns of behaviour ('demi-regularities'[14]) occur. Realist review seeks to uncover the underlying theories that explain these demi-regularities by critically scrutinising the interaction between context, mechanism and outcome in a sample of primary studies. Mechanisms are processes operating within an intervention that describes how the 'human components' use the resources available to them [14,15]. In particular middle-range theory (that is theory that "involves abstraction... but [is] close enough to observed data to be incorporated in propositions that permit empirical testing."[16]) is specifically sought as their level of abstraction provides a more generalisable explanation of demi-regularities. More than one middle-range theory may explain the influences of context on a mechanism to produce an outcome [14].

Importantly, realist review methodology acknowledges that within complex interventions there are many dimensions and layers of explanation that warrant exploration. For example, there are human behaviours as well as multiple interactions between the numerous components of the intervention. A realist review does not seek to explain all these layers; it is specifically focused on the demi-regularities in the social (and socio-technical) world which create preconditions for particular human behaviours [14]. To that end, we sought to extract theories from our dataset of primary studies which would explain whether or not an Internet based course was considered a 'success' and especially whether it produced effective learning. We sought to try to gain insights and explanations that would be generalisable across a whole range of different types of Internet based courses and so theories that focused on specific aspects of such courses (for example only computer mediated conferencing) were not central to our inquiry.

### Inclusion and exclusion criteria

Studies were included if they had any medical students or doctors as learners; used the Internet to support learning; and contained at least one level of evaluation as described by Kirkpatrick [17]. Studies were excluded if the Internet was used for purposes other than learning (e.g. tracking website use, examinations only, course administration).

### Identifying primary studies

We searched 15 electronic databases relevant to medical education from their inception dates to April 2006 using guidance provided by Haig and Dozier [18,19]. No language restrictions were applied (non-English language papers were translated), and publications of any type were included. Details of the databases and search strategy are available in Additional file 1.

In the first stage of searching, GW screened the title, abstract and subject headings (where available) against inclusion and exclusion criteria. Potentially eligible studies were obtained in full text and re-screened in the second stage. A random subset (200/12586 and 50/514 citations respectively) at each stage was screened independently by TG and disagreements resolved by discussion.

### Identifying candidate theories

The initial identification of candidate (middle-range) theories in realist reviews is necessarily an iterative and speculative process. Whilst a review team may initially have theories that they believe to be in operation to explain why certain outcomes occur, a key element in realist review is to explore the presence of these 'educated guess' theories and where applicable, test their explanatory value. Candidate theories are not considered definitive until they have been tested. Much of the work in realist review involves not only repeatedly questioning the validity of any candidate theory and refining it but also seeking out new candidate theories from included studies if existing ones are found wanting.

We used a variety of methods to derive our list of candidate theories. This included; brain-storming within the review team, browsing through specialist educational library collections, discussions with fellow educators and pursuing references of references [20]. We did not specifically consult individual experts in the field. We iteratively [re-]checked all the included studies against the candidate theories so as to establish which (if any) explained differences in outcomes. In each paper, we sought data to test (affirm, refute or refine) the candidate theory by assessing their relevance and rigour [14]. Throughout our data extraction and synthesis phases, we continually sought out further candidate theories that might better explain the data in the included studies.

### Data management, analysis and synthesis

In a first phase, study characteristics (e.g. sample type and size, setting, course objectives) and theoretical contribution (e.g. 'how', 'why, 'in what circumstances') were tabulated on an Excel spreadsheet using data domains informed by previous systematic reviews in this field [2,5,6,21]. In a second phase, the NVivo qualitative software was used to index and link relevant sections of text of included articles to our emerging analytic framework [22]. As each included article was read and re-read, we created and iteratively revised codes to capture themes or concepts that might contribute to theory testing [23]. In particular, we sought to identify prominent demi-regularities that might help us to understand Internet-based interventions better. We classified 'interaction' in the online environment according to the criteria of Vrasidas and Glass (in sum, learner-tutor, learner-learner, learner-content and learner-software, the last of these being technical feedback such as automated replies to multiple choice questions) [24].

Data synthesis involved both individual reflection and team discussions that considered the ability of the candidate theories to explain the data reported in empirical studies (especially in relation to any prominent demi-regularities we encountered). The sections of texts from our included studies, which we had coded and captured within NVivo formed the raw materials for our interpretations. We used these sections of texts to see if they were able to confirm, refute or refine our candidate theories. Specifically, we attempted to identify recurrent demi-regularities which might act as barriers or enablers to Internet-based learning and tested the explanatory powers of our initial candidate theories against these. Where candidate theories failed to explain the data we sought new ones, either from the included studies or wider educational or sociological literature. Throughout this process, we deliberately sought out disconfirming data - i.e. data that might refute our provisional candidate theories. In line with realist review methodology, we also used the information we gleaned from our immersion in our included studies to refine our initial review goals [14].

## Results

### Search results and study characteristics

**Figure 1:**
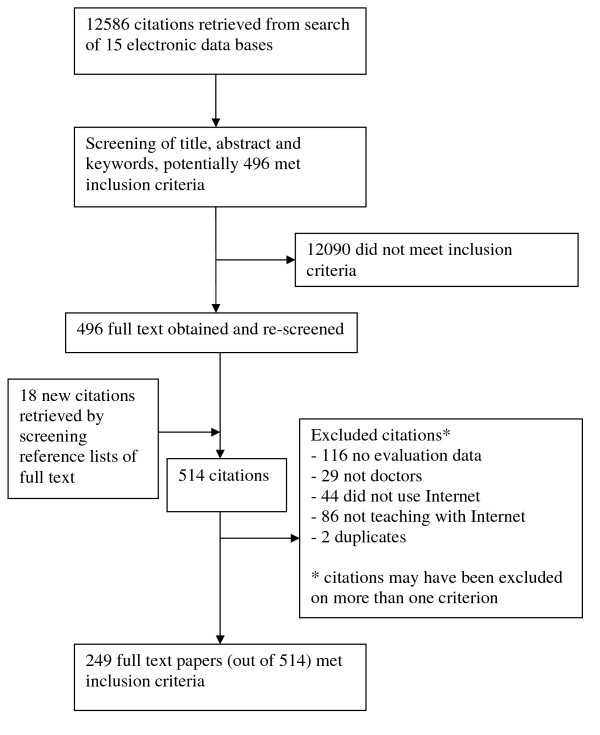
**Flow chart of screening process**. This figure outlines how we arrived at the 249 full text papers we included in our realist review.

Figure 1 shows the numbers of included studies at each stage of the review. The raw inter-rater agreement for inclusion/exclusion was 92% (183/200) in the first stage and 84% (42/50) in the second stage. The 249 articles were published in 133 different journals and included a total of 44,591 participants. In all, 20% (49/249) of studies were randomised trials; 66% (165/249) non-randomised controlled studies (usually controlled before and after studies); 7% (18/249) mixed methods and 7% (17/249) not stated. When compared against the study's aim(s) or objective(s), 72% (179/249) reported positive outcomes and 22% (55/249) had mixed findings. In terms of Kirkpatrick's levels of evaluation, 84% (209/249) of studies measured learner satisfaction; 50% (124/249) learning outcomes; 3% (7/249) behaviour change and 0.4% (1/249) patient outcomes.

### Candidate theories

We initially selected four candidate theories for further testing: Laurillard's conversational framework [25], Schon's reflective practitioner [26], Slotnick's how doctors learn [27] and Reeves' effective dimensions of interactive learning [28].

These theories provided only a *starting point *in our attempt to explain what sort of Internet-based medical education 'works', for whom and in what circumstances. As we extracted our data, we noted further candidate theories and proceeded to test these as well. Additional candidate theories that we attempted to tested included: Vygotski [29], Danchak [30], Schon [31], Garrison [32,33], Dewey and Brookfield [33], Kolb [34], Moshman [35], Eraut [36], Boettcher [37], Wenger [38], Koschmann [39], Nahapiet and Ghoshal [40], Socrates [41] Problem Based Learning [31,42-48], Constructivism [29-31,33-35,37,44,45,49-57] and adult learning theory/principles [31,32,47,50,53,54,58-70].

As no previous realist review had been undertaken in this field, we were initially unclear as to how suitable the data reported in our included studies would be for answering the broad research question goal we had set ourselves. As the review progressed we became aware of various data suitability limitations (see Discussion) and the emergence of two prominent demi-regularities prompted us to narrow our review focus to the two candidate theories discussed below. This is an example of progressive focusing - a well-established technique in qualitative research in which the focus of the inquiry is iteratively sharpened by reflection on emerging data [71].

### Technology acceptance: getting learners to log on

At an early stage in this review, our reading and interpretation of the reported data in our included studies showed that educators often faced a substantial barrier of getting learners to use their Internet-based course. This demi-regularity of getting learners to log onto - or engage with - a course was clearly an important factor in explaining the fortunes of such courses. We noted that learners needed to have to have good reasons to engage and that unless they did, the outcomes reported were less favourable. Examples of the texts we used to support our interpretation may be found in Additional file 2: Table s1.

Engagement and acceptance was not explained by any of our initial candidate theories, but we noted that one of our included papers [72] mentioned the value of conceptualising Internet based courses as innovations and specifically Rogers' diffusion of innovations theory [73]. We found that Davis's Technology Acceptance Model [74], which is derived from Rogers' theory, was a more precise articulation of innovation acceptance when the innovation involved was a technology. Drawing on both Rogers' and Davis's theories, the attribute of an Internet-based course that provided the most coherent and complete explanation of technology acceptance was the perceived usefulness of the technological medium (in the eyes of potential learners) over an alternative delivery format. From our included studies, we identified that perceived usefulness - or in Rogers' original terminology 'relative advantage', included 7 sub-components, representing the contexts that influence whether learners choose to engage with an Internet-based course: access to learning; access to consistent content; links with assessment; convenience; cost saving; interactivity; and time saving.

Overall, 38% (95/249) of our included studies provided some data to support the central importance of perceived usefulness and none provided data to refute it. Two other attributes - perceived ease of use (from Davis's Technology Acceptance Model) and compatibility with the learner's norms and values (from Rogers' original diffusion of innovations theory), also explained some of the variability in acceptance of the Internet medium, and evidence to support these attributes was found in 13% (32/249) and 3% (7/249) of studies respectively. Again, we found no disconfirming studies.

We wanted to provide a set of recommendations that would help course developers and learners make of most of an Internet based course. Thus we converted the three attributes within Davis's Technology Acceptance Model that we were able to test - perceived usefulness, perceived ease of use and compatibility - into three questions (one of which included seven sub-questions, representing the important contextual influences), which are shown in Table 1.

**Table 1:** Five questions for developers and prospective learners to ask of an Internet-based course

Technology acceptance	
1.	How useful will the prospective learners perceive the Internet technology to be?
	For example, in any particular context and compared to what is currently available to them, to what extent will this technology
	a. Increase their access to learning?
	b. Provide consistent, high-quality content?
	c. Be a convenient format in which to receive their education?
	d. Save them money?
	e. Save them time?
	f. Link to course assessment?
2.	How easy will the prospective learners find this technology to use?
3.	How well does this format fit in with what learners are used to and expect?
**Achieving interactive dialogue**	
4.	How will high-quality human-human (learner-tutor and learner-learner) interaction and feedback be achieved? For example what use will be made of
	a. Structured virtual seminars?
	b. Email, bulletin boards?
	c. Real-time chat?
	d. Supplementary media e.g. video, audio, phone calls, videoconferencing?
	e. Course assessment and feedback on performance?
5.	How will high-quality human-technical interaction and feedback be achieved? For example what use will be made of
	f. Questions with automated feedback?
	g. Simulations?

### Interaction: building a learning dialogue

**Figure 2:**
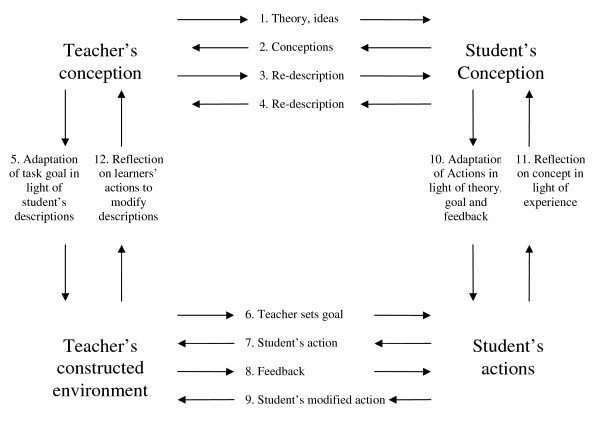
**Laurillard's Conversational Framework**. This figure is a diagrammatic representation of the all the stages that go to make up the dialogue between a teacher and a student.

The primary studies frequently reported that learners greatly valued courses that allowed them to 'interact' - though this term was rarely defined. This demi-regularity was consistent across different course designs and other characteristics (e.g. participant type, age, gender). Laurillard's Conversational Framework (Figure 2) was the middle-range theory that explained these data particularly well [25]. This theory is built on the assumption that a learner learns by entering into a dialogue with others (virtual or human) in order to clarify understanding and obtain feedback on performance. Overall, 36% (90/249) of included studies provided some data which supported (and none provided data that refuted) the Conversational Framework. Examples of the texts we used to support our interpretation for the Conversational Framework may be found in Additional file 2: Table s2.

In our recommendations in Table 1, we have again converted our insights about the importance of interaction and feedback into two questions which remind course developers to think about this issue. The examples that we have provided of how the interaction and feedback might be enabled technically are drawn from our analysis of the methods used in our included studies.

### Course-context interaction

An important finding of this review was that 'success features' did not seem to be intrinsic to any course but a function of the course-context interaction. One group of learners might perceive the a technologically based course as having very high 'usefulness' while a different group would find the same course much less useful. For example, in studies comparing virtual microscopy (where glass slides were digitised and the features of a traditional light microscope simulated by software) with conventional microscopy, medical student learners were reported as valuing the Internet-based materials much more highly and utilised these more. Features of perceived usefulness included assessment linkage (virtual material was used in exams) [75], consistent high quality content (whereas traditional slides may or may not show the feature concerned) [76]; convenience (they did not have to conform to laboratory opening times) [77]; cost saving (rental cost of a microscope) [76]; and time saving (journey times to the laboratory were cut to zero) [77]. The course's ease of use (comments included "doesn't hurt my eyes" "stays in focus") was also highly rated compared to conventional alternatives [78]. However, this same Internet-based application was reported as having little or no perceived usefulness for trainee pathologists, who must learn not merely to evaluate standardised slides in formal examinations but to deal with the inconsistencies and contextual complexities of real slides in the real world [79].

The above example also suggests that the construct 'ease of use' does not operate independently of other course features, especially its perceived usefulness. For example, we encountered studies utilising virtual textbooks (where text and/or images were digitised and placed online) where despite efforts to ensure the technology was easy to use, learner engagement remained low (e.g. because the learners perceived that they could access 'better' but similar content face to face or in other formats) [80-83]. Conversely, we found a 1996 paper describing a bio-computing course that had been set up to allow teaching expertise to be shared between the few geographically dispersed experts there were in this field [84]. The tutors and highly computer literate students communicated using a very rudimentary and technically complicated email system. Despite these challenges, most students persisted with it and rated their learning experience as positive. It appears that the advantage of being able to learn with otherwise hard-to-reach experts ('improved access to learning') more than made up for the technical limitations of the learning technology.

## Discussion

### Summary of main findings

This realist review of 249 primary studies has produced two key findings which are important if somewhat unsurprising. First, Internet-based courses must engage their target group of learners to use the technology. This is likely to occur only if the technology is perceived as 'useful' (e.g. increases access to learning or saves time) and 'easy to use', though benefits in the former can outweigh challenges in the latter. Second, 'interactivity' is highly valued by learners. Learners wanted to be able to enter into a dialogue with the course tutor, fellow students and/or a virtual tutorial and obtain ongoing feedback on their understanding and performance.

Course design is an important factor in Internet-based courses, but attention must also be paid to course-context interaction. A pedagogically sound course may prove technically acceptable and produce positive learning outcomes in one group of learners in one context but the *same *course may be technically unacceptable and/or fail to achieve effective learning in a different context. The skills of learners, course learning objectives and the availability, quality and cost of non-Internet alternatives are particularly important contextual factors.

### Strengths and limitations of the review

To our knowledge, this review represents the first use of realist review in medical educational research. It contributes to an emerging field in systematic review, in which qualitative reviews are undertaken to supplement and extend the findings of meta-analyses and other quantitative reviews [85,86]. The advantage of using both approaches is that the strengths and weaknesses of each method are complementary [87-89]. Realist reviews are a type of theory driven qualitative review and so differs in many respects to more quantitative (for example Cochrane) reviews. A discussion of the advantages and disadvantages between these review methods is beyond the scope of this paper and interested readers are directed to Chapter 3 of Pawson's Evidence-based Policy: A Realist Perspective [14].

The recent meta-analysis by Cook et al (see Background) provided much-needed evidence that the overall educational impact of Internet-based medical education can be equivalent to that of conventional formats. In their discussion, these authors raised two further questions which they acknowledged had not been addressed by their meta-analysis: "*How *can Internet-based learning be effectively implemented?" and "*When *should Internet-based learning be used?" [8]. Cook has previously observed that "...the appropriateness of web-based learning as a learning tool will vary upon the instructional context..." - a comment which raises the question of what sort of course is 'appropriate' in what sort of context [90].

Our review has begun to extend the knowledge base by identifying and refining some of the middle-range theories that explain the 'how', 'why' and 'in what circumstances' questions about Internet-based medical education. We acknowledge that our progressive focus on two prominent demi-regularities has meant that we have not addressed all aspects of our initial review's goals. However, it is reassuring that the key findings of this review align with, and illuminate, the findings of previous systematic reviews. For example, the *quantitative *observation that the speed of downloading is associated with learner satisfaction [21] may be explained *qualitatively *by the 'ease of use' construct within the Technology Acceptance Model (and, more widely, diffusion of innovation theory). Similarly, the observation that 'dialogue' [4] and interaction [91] is associated with improved learner performance is explained qualitatively by the Conversational Framework.

Perhaps more significantly, theory-driven qualitative systematic reviews may also throw light on the reason why there is a *lack of *association between variables and outcomes seen in quantitative (Cochrane-type) reviews. We suggest that a paradigm shift may need to occur in how interventions that involve human agency should be viewed - namely as complex interventions [12,13].

The pursuit of rigour in realist review follows similar principles to the pursuit of rigour in qualitative research more generally [92]. The essence of such research is interpretation, hence key processes are immersion (reading and re-reading texts), reflection, discussion amongst team members, comparison and continuing to seek explanations and test theories until saturation of the data is reached. Our sample included a heterogeneous group of primary studies of different learner groups in diverse contexts, with no restrictions by study design or language of publication - in other words, we had what is known in qualitative research as a 'maximum variety sample'. This allowed us to explore a wide range of context-mechanism-outcome combinations and use the available qualitative data reported in the primary studies to build and refine theories of how Internet-based learning 'works'. Whilst we have followed the realist review method and documented the steps we took to arrive at the middle-range theories presented here, we are fully aware that (in common with other qualitative research) this method is subjective and interpretive. Therefore another team reviewing the same literature may arrive at a different set of middle-range theories with which to make sense of this vast field.

We did not consult individual experts in this field and acknowledge that had we done so, we may well have had a wider set of additional candidate theories to test. We did not set out to be all-inclusive in our review but have been able to uncover key middle-range theories that begin to help to explain the fortunes of Internet based courses. We are certain that other middle-range theories will be needed and are important in furthering understanding and believe that there is more work to be done in unravelling the multitude of theories that are in operation within Internet based courses. More specifically, we believe that more theory drive reviews, such as ours and that by Ruiz et al. [93] hold the greatest promise to understanding medical educational interventions.

Whatever review method is used in secondary research, the resulting synthesis is only as good as the primary data on which the synthesis is built. A major limitation we encountered in our review was that many primary studies included only cursory descriptions of their Internet-based educational intervention (e.g. educational setting, teaching practices and rationale of course design). The paucity of such data placed two important limitations on our review. Firstly, we were not able to test in detail all aspects of our candidate theories. If richer descriptions been reported in our included studies, we would have been able to undertake a more fine-grained analysis of both technology acceptance and interactivity. Secondly, we were aware that a large number of theories exist on how learners learn online and in more traditional settings. In our included studies alone, 17 specific theories were named in 58 articles. However, within the included studies, we could not find sufficient reported detail to enable comprehensive testing of these theories.

Limitations in the type of data, depth and quality of reporting of studies in medical education are well recognised [94]. We strongly recommend that authors of primary studies in this field produce detailed descriptions of the intervention and context *as well as *quantitative data on satisfaction and impacts, and that journal editors make space for these rich descriptions, since the ability of future realist and other theory driven reviews to extend the knowledge base further will depend on the quality and completeness of the qualitative data gathered and reported.

## Conclusions

Based on the findings of this review we suggest a set of questions that educators should address in order to maximise the chance that their Internet-based courses will be perceived as useful and provide an effective learning opportunity, and which prospective learners may use to evaluate whether a course is right for them (Table 1). Given our findings above about the importance of course-context interactions, it follows that the factors referred to in Table 1 cannot be 'built into' courses independently of a consideration of learners' needs and priorities or assessment of other courses available locally and indeed, on the Internet - in other words the course's context. Nor can our guidance be seen as a deterministic 'law of nature' which if slavishly followed will invariably lead to a successful course. The questions in Table 1 are designed to complement existing guidance on course design (such as for example by Grant [95] or McKendree [96]), and should be seen as part of the entire curriculum design process and not as a substitute for these.

## Competing interests

The authors declare that they have no competing interests.

## Authors' contributions

GW had the original research idea and it was refined with the help of TG and RP. GW carried out all stages of the review. TG independently screened a sample of articles for inclusion. TG and RP oversaw the data extraction and synthesis stages. GW drafted the paper and TG and RP both contributed significantly on the overall content, concepts and structure of subsequent drafts. All authors have read and approved the manuscript and GW is the guarantor of this paper.

## Pre-publication history

The pre-publication history for this paper can be accessed here:

http://www.biomedcentral.com/1472-6920/10/12/prepub

## Supplementary Material

Additional file 1 **Databases searched and search strategy**. This file contains a list of all the data bases we searched and an example search strategy indicating the terms we used. Click here for file

Additional file 2 **Verbatim examples of sections of texts used in data synthesis**. This file contains illustrative examples of verbatim text drawn from our included studies that were used to test Davis's Technology Acceptance Model (Table s1) and Laurillard's Conversational Framework (Table s2). Click here for file
