# Supplementary material for: Internet-based medical education: a realist review of what works, for whom and in what circumstances
Source: BMC Med Educ. 2010 Feb 2;10:12. doi: 10.1186/1472-6920-10-12 (PMC2825237; doi:10.1186/1472-6920-10-12)
Supplement: Additional file 1 — Databases searched and search strategy. This file contains a list of all the data bases we searched and an example search strategy indicating the terms we used. [file 1472-6920-10-12-S1.DOC]

### Additional file 1 - Databases and search strategy

The following electronic databases were searched:

- Medline
- EMBASE
- CINAHL
- Education Resource Information Center (ERIC)
- PsycINFO
- British Education Index (BEI)
- British Education Internet Resource Catalogue
- Education *on-line*
- Research and Development Resource Base (RDRB)
- TIMELIT
- Cochrane Library (includes; Cochrane Controlled Trial Register (CCTR); Cochrane Database of Systematic Reviews (CDSR) and Database of Abstracts of Reviews of Effect (DARE))
- Health Management Information Consortium (HMIC)
- Dissertation Abstracts
- Organisation for Economic Co-operation and Development (OECD) publications
- ISI Web of Science

For each database searches were based on using subject headings (where possible) and related free text terms (where possible with truncations and proximity operators). The main search terms were broad and covered *participants*, *educational intervention type* and *outcomes* and these were combined with the AND Boolean operator. The following broad generic terms were used and combined with the OR Boolean operator:

*Participants*:

MEDICAL EDUCATION, MEDICAL TEACHING, MEDICAL INSTRUCTION, MEDICAL LEARNING, UNDERGRADUATE MEDICAL, EDUCATION/TEACHING/INSTRUCTION/LEARNING, POSTGRADUATE MEDICAL EDUCATION/TEACHING/INSTRUCTION/LEARNING, EDUCATION

*Educational intervention type*:

INTERNET, WEBBASED, WEB-BASED, WEB BASED, ONLINE, ON-LINE, ON LINE, COMPUTER ASSISTED LEARNING, COMPUTER-ASSISTED-LEARNING, COMPUTER ASSISTED TEACHING, COMPUTER-ASSISTED-TEACHING, COMPUTER ASSISTED INSTRUCTION, COMPUTER-ASSISTED-INSTRUCTION, COMPUTER AIDED LEARNING, COMPUTER-AIDED-LEARNING, COMPUTER AIDED TEACHING, COMPUTER-AIDED-TEACHING, COMPUTER AIDED INSTRUCTION, COMPUTER-AIDED-INSTRUCTION, DISTANCE-LEARNING, DISTANCE-LEARNER/S

*Outcomes*:

CONTROLLED TRIAL, QUASI EXPERIMENTAL, QUESTIONNAIRE, EVALUTION, ASSESSMENT, APPRAISAL
