# Supplementary material for: Internet-based medical education: a realist review of what works, for whom and in what circumstances
Source: BMC Med Educ. 2010 Feb 2;10:12. doi: 10.1186/1472-6920-10-12 (PMC2825237; doi:10.1186/1472-6920-10-12)
Supplement: Additional file 2 — Verbatim examples of sections of texts used in data synthesis. This file contains illustrative examples of verbatim text drawn from our included studies that were used to test Davis's Technology Acceptance Model (Table s1) and Laurillard's Conversational Framework (Table s2). [file 1472-6920-10-12-S2.DOC]

### Additional file 2 - Verbatim examples of sections of texts used in data synthesis

**Table s1: Examples of text used to support our interpretations for the Technology Acceptance Model**

| **Perceived usefulness** |
| --- |
| *Access to learning* |
| “Nova Scotia is well suited to evaluating [Internet mediated] video-conferencing for practice-based small-group CME. Through the Nova Scotia Telehealth Network, Dalhousie CME has provided regular videoconferenced CME programs since 1997. Many communities that receive videoconferenced CME have three or fewer physicians who could benefit from linking with colleagues in similar sites for small-group learning.” [1]  “Nevertheless, the preceptors felt that this type of presentation allowed better use of limited resources. It enabled them to reach a broader audience with nominal extra time. They felt that it was most beneficial to the site B (distant teaching site) residents, who may experience more difficulties accessing these teaching sessions.” [2] |
| *Access to consistent course content* |
| “CAI has therefore provided several students with a welcome relief from what may be frustrating and tedious laboratory sessions, where structures are frequently misidentified.” [3]  “The advantages for group work were repeatedly mentioned (28 respondents), with comments including: ‘Great because everyone sees the same image and people aren’t staring down a microscope — so are more able to talk’, ‘I enjoyed it because you and your partner were looking at the same slide so it was easier to talk about it’, ‘Virtual slides provide a very clear image, easier to use and to discuss. Easier to ask questions and good for interactive learning’” [4] |
| *Linkage with assessment* |
| “The integration of e-learning into the curricular mainstream also played a pivotal role for the virtually 100% rate of voluntary utilization of k-MED material by the students.”[5]  “Without the extra incentive of CME credits and the tedious requirement of the evaluation instrument, only the diehard and those very keen have enrolled in the study.”[6] |
| *Convenience* |
| “A number of students also commented on the advantages of access from home (if they had a broadband Internet connection) for revision.”[4]  “Students recognise the opportunities are offered in virtual reality, including the practice of technical skills and the chance to make mistakes in a safe environment without harm to the patient.”[7] |
| *Cost saving* |
| “Rural physicians thought this delivery mode would enable them to participate in distance CME, regardless of geography or time. This would mean significant cost savings, because they would not have to travel long distances, secure replacement locums, or travel away from family.”[8]  “In addition, the e-Textbook saved students the expense of purchasing a textbook for a short 2-week clerkship.”[9] |
| *Interactivity* |
| See Table 2 |
| *Time saving* |
| “Removal of barriers to attendance at traditional CME, particularly lost time from practice and family and distance to travel, was valued. This was most marked for rural physicians.”[10]  “Faculty generally expressed enthusiasm for using the technology to present CME programs to physicians in distant parts of the province. One comment was “This is so much easier to do than travel.””[10] |
| **Perceived ease of use** |
| “The ease of use of the software was rated extraordinarily highly; open-ended comments in response to the question about this included: ‘Ridiculously easy. A 4-year-old (with weird interests) could do it’, ‘Very user-friendly’, and ‘Very straightforward.’”[4]  “Students prefer CAI to traditional glass slides because the skill level and the time required for learning CAI is less than the time needed to use a microscope or projector.” [11] |
| **Compatibility** |
| “Face-to-face contact is important; I want to be taught by a teacher, not a machine.”[12]  “Residents thought that the distance learning technology was implemented effectively and viewed distance learning as an important method for delivering didactic conferences. Residents recorded that they would recommend distance learning to other residents, and they believed that distance learning methods are as effective as traditional classroom methods for the delivery of didactic topics. While the residents felt that the distance learning site was equal to the traditional classroom, they did not prefer it.” [alternatives] [13] |

**Table s2: Examples of text used to support our interpretations for Laurillard’s Conversational Framework**

| “The interactive dialog probably played a large role in the program’s effectiveness by encouraging the students to work through problems, inducing them to take more time on particular tasks and probably to give more attention to the material.”[14]  “When asked to design an effective learning tool for this e-course, the students unanimously said they wanted interactivity—interactive quizzes and interactive cases…. Students want e-courses to be built around cases, quizzes, and conversations with teachers.”[15]  “After surveying the students, the modular and dynamic teaching approach of simPHYSIO (interactivity, user driven manipulations, animations) was one of the most significant features in helping the student learn the material and enjoy the learning process…… “I like the interactive approach of virtual labs. It made the concepts easier to understand.””[16]  “The conclusion that we draw from the quantitative and qualitative data is that while students enjoyed the opportunity to quiz themselves, they would have benefited from more guidance in the form of elaborate feedback on the correct and incorrect answers to questions. The comments also show that students would have liked more instruction in the case-based tutorials in the virtual laboratory.”[17]  “…students cited insufficient feedback in the "Self-Assessment" module as another problem. The only feedback to students was their score on the correctly identified descriptors out of the total possible score. Students were dismayed by not knowing what the correct answers were and why their own responses were considered inaccurate. This led them to engage in protracted and frustrating efforts to maximize their score with repeated access to the module. For example, several students reported that they took one exam item at a time, downloaded the score, and repeated the process until they found a set of descriptors that maximized their score.”[18] |
| --- |

Reference List

1. M Allen, J Sargeant, K Mann, M Fleming, J Premi: **Videoconferencing for practice-based small-group continuing medical education: feasibility, acceptability, effectiveness, and cost.** *J Contin Educ Health Prof* 2003, **23:** 38-47.

2. KI Kroeker, I Vicas, D Johnson, B Holroyd, PA Jennett, RV Johnston: **Residency training via videoconference--satisfaction survey.** *Telemed J E Health* 2000, **6:** 425-428.

3. M McLean: **Web pages: an effective method of providing CAI resource material in histology.** *Med Teach* 2001, **23:** 263-269.

4. RK Kumar, GM Velan, SO Korell, M Kandara, FR Dee, D Wakefield: **Virtual microscopy for learning and assessment in pathology.** *J Pathol* 2004, **204:** 613-618.

5. M Gotthardt, MJ Siegert, A Schlieck, S Schneider, A Kohnert, MW Gross, C Schafer, R Wagner, S Hormann, TM Behr et al.: **How to successfully implement e-learning for both students and teachers.** *Acad Radiol* 2006, **13:** 379-390.

6. DH Chan, K Leclair, J Kaczorowski: **Problem-based small-group learning via the Internet among community family physicians: a randomized controlled trial.** *MD Comput* 1999, **16:** 54-58.

7. DC Alverson, SMJ Saiki, J Jacobs, L Saland, MF Keep, J Norenberg, R Baker, C Nakatsu, S Kalishman, M Lindberg et al.: **Distributed interactive virtual environments for collaborative experiential learning and training independent of distance over Internet2.** *Stud Health Technol Inform* 2004, **98:** 7-12.

8. VR Curran, T Hoekman, W Gulliver, I Landells, L Hatcher: **Web-based continuing medical education. (II): Evaluation study of computer -mediated continuing medical education.** *J Contin Educ Health Prof* 2000, **20:** 106-119.

9. CS Jao, SU Brint, DB Hier: **Making the neurology clerkship more effective: Can e-Textbook facilitate learning?** *Neurol Res* 2005, **27:** 762-767.

10. DB Langille, JM Sargeant, MJ Allen: **Assessment of the Acceptability and Costs of Interactive Videoconferencing for Continuing Medical Education in Nova Scotia.** *J Contin Educ Health Prof* 1998, **18:** 11-19.

11. Klatt E: **Web-based teaching in pathology.** *JAMA* 1997, **278:** 1787.

12. M Cannon, K Umble, A Steckler, S Shay: **We're Living What We're Learning": Student Perspectives in Distance Learning Degree and Certificate Programs in Public Health.** *J Public Health Manag Pract* 2001, **7:** 49-59.

13. T Markova, LM Roth, J Monsur: **Synchronous distance learning as an effective and feasible method for delivering residency didactics.** *Fam Med* 2005, **37:** 570-575.

14. JA Grundman, RS Wigton, D Nickol: **A controlled trial of an interactive, web-based virtual reality program for teaching physical diagnosis skills to medical students.** *Acad Med* 2000, **75:** S47-S49.

15. JD Hoban, JB Schlesinger, RP Fairman, MM Grimes: **Electrifying a medical school course: a case study.** *Teach Learn Med* 2003, **15:** 140-146.

16. C Huang: **Changing learning with new interactive and media-rich instruction environments: virtual labs case study report.** *Comput Med Imaging Graph* 2003, **27:** 157-164.

17. AM Marchevsky, A Relan, S Baillie: **Self-instructional "virtual pathology" laboratories using web-based technology enhance medical school teaching of pathology.** *Hum Pathol* 2003, **34:** 423-429.

18. GJ Raugi, S Kim, PB Odland: **Teaching morphology on the world wide web: The experience of "Language of Dermatology".** *Dermatol Online J* 1996, **2:** 1-12.
